# Primary HIV infection: the French experience

**DOI:** 10.1186/1742-4690-9-S1-I14

**Published:** 2012-05-25

**Authors:** Cécile Goujard

**Affiliations:** 1 General Hospital of Bicêtre, Kremlin-Bicêtre, France

## 

The ANRS PRIMO is a prospective multicenter French Cohort which had enrolled 1433 patients during primary-HIV-1 infection (PHI) since 1996, with the objectives to improve pathophysiological knowledge on PHI, to assess the impact of an early antiretroviral treatment and to contribute to document the epidemiology of new infections in France. Clinical and laboratory data are collected at inclusion, month (M)1, M3, M6, and then every 6 months. All patients are antiretroviral-naïve at inclusion.

Patients were included early after infection (median time, 31 days; 26% acute infection); 84% were men. The % of patients infected with non-B subtypes increased in the last decade, while the frequency of resistant viral strains remained stable. At inclusion, median CD4 cell counts and viral HIV load were 518/mm^3^ and 5.1 log_10_ copies/mL at PHI, with a wide range of values in the whole population (IQR, 372-678/mm^3^ and 4.5-5.7 log respectively), depending only partially to the time from infection. Median cellular DNA at inclusion was 3.4 log cp/10^6^ PBMCs (IQR, 2.9-3.7). Early immunological (CD4 counts) and virological parameters (HIV RNA and DNA) could predict the risk of progression to a CD4 count < 350 cells/mm^3^ in untreated patients and the chance of spontaneous persistent control of viral replication after infection. 52% of patients initiated an antiretroviral treatment at inclusion. Although a transient treatment during PHI did not translate into a benefit in terms of viral set-point compared to untreated patients, it could lead to long term preservation of CD4 cell counts. Furthermore, we showed that rare treated patients were capable of controlling viral replication after treatment interruption, those patients had lower viral reservoir at inclusion before treatment and at treatment interruption compared to non controllers. Cellular activation was high during PHI and declined substantially thereafter, with lower levels in patients treated early after infection compared to patients treated during the chronic phase.

Antiretroviral treatment is now given in less advanced infection and even discussed as a universal approach to prevent HIV transmission. The question of treatment initiation in all patients diagnosed during PHI is relevant.

